# Strength Training Prevents Hyperinsulinemia, Insulin Resistance, and Inflammation Independent of Weight Loss in Fructose-Fed Animals

**DOI:** 10.1038/srep31106

**Published:** 2016-08-04

**Authors:** José D. Botezelli, Andressa Coope, Ana C. Ghezzi, Lucieli T. Cambri, Leandro P. Moura, Pedro P. M. Scariot, Rodrigo Stellzer Gaspar, Rania A. Mekary, Eduardo Rochete Ropelle, José Rodrigo Pauli

**Affiliations:** 1 Department of Nutrition, Metabolism and Exercise, Campinas State University (UNICAMP), Limeira/SP, Brazil; 2 Medical Sciences University, Campinas State University (UNICAMP), Campinas/SP, Brazil; 3 Department of Physical Education, Mato Grosso Federal University (UFMT), Cuiabá/MT, Brazil; 4 Department of Pharmaceutical Business and Administrative Sciences; MCPHS University, Boston/MA, USA; 5 Department of Surgery; Brigham and Women’s Hospital, Harvard Medical School, Boston/MA, USA

## Abstract

The aim of this study was to compare the effects of aerobic, strength, and combined training on metabolic disorders induced by a fructose-rich diet. Wistar rats (120 days old) were randomized into five groups (n = 8–14): C (control diet and sedentary), F (fed the fructose-rich diet and sedentary), FA (fed the fructose-rich diet and subject to aerobic exercise), FS (fed the fructose-rich diet and subject to strength exercise), and FAS (fed the fructose-rich diet and subject to combined aerobic and strength exercises). After the 8-week experiment, glucose homeostasis, blood biochemistry, tissue triglycerides, and inflammation were evaluated and analyzed. The strength protocol exerted greater effects on glucose homeostasis, insulin sensitivity, and liver lipid contents than other protocols (all P < 0.05). All three exercise protocols induced a remarkable reduction in inflammation, tissue triglyceride content, and inflammatory pathways, which was achieved through c-Jun NH2-terminal kinase (JNK) phosphorylation and factor nuclear kappa B (NFkB) activation in both the liver and the muscle. Our data suggest that strength training reduced the severity of most of the metabolic disorders induced by a fructose-rich diet and could be the most effective strategy to prevent or treat fructose-induced metabolic diseases.

Metabolic syndrome (MS) is a world-wide and growing problem^1^ that is associated with an excessive intake of sugar and fat^2^ and a decrease in daily energy expenditure^3^. The average prevalence of MS in the United States is 34% in adults and 50–60% in older adults (>60 years); thus, MS constitutes a major public health problem and an economic burden^4^,^5^. A state of chronic inflammation occurs with MS and may be the cause of increased dependence on lipids as an energy source, insulin resistance, subsequent impaired signaling, and cell structure damage, which lead to a vicious cycle^6^,^7^. Obesity is a well-known risk factor for metabolic diseases. Most strategies for treating and preventing non-communicative metabolic diseases involve weight loss and adipose tissue reduction. The American College of Sports Medicine (ACSM) recommends at least 150 minutes per week of moderate-intensity exercise and 2–3 days per week of resistance training to reduce body weight and prevent metabolic diseases^8^. Despite the importance of the ACSM guidelines, these recommendations are far from being successful, given the yearly progressive decline in time spent in physical activity^9^,^10^. In this context, new exercise protocols have been suggested to treat metabolic disorders without necessarily losing body weight^11^,^12^,^13^. Our group published many studies showing the underlying physiological and molecular effects of different aerobic exercise regimens on several metabolic diseases^14^,^15^,^16^; there is, however, new evidence that strength exercise could produce significant improvements in health biomarkers and could even treat or prevent metabolic diseases^17^,^18^. To our knowledge, no previous study has compared different exercise modalities (aerobic, strength, or combined training) on metabolic disorders induced by a fructose-rich diet in rats.

In this study, we compared the effects of aerobic, strength, and combined training on insulin sensitivity, glucose homeostasis, dyslipidemia, and inflammation with minor or no changes in the body weight of Wistar rats. To achieve such a deleterious metabolic environment with no changes in body weight, we used fructose, a common nutrient present in Western diets^14^,^19^.

## Results

### Minimum lactate test

**Figure 1:**
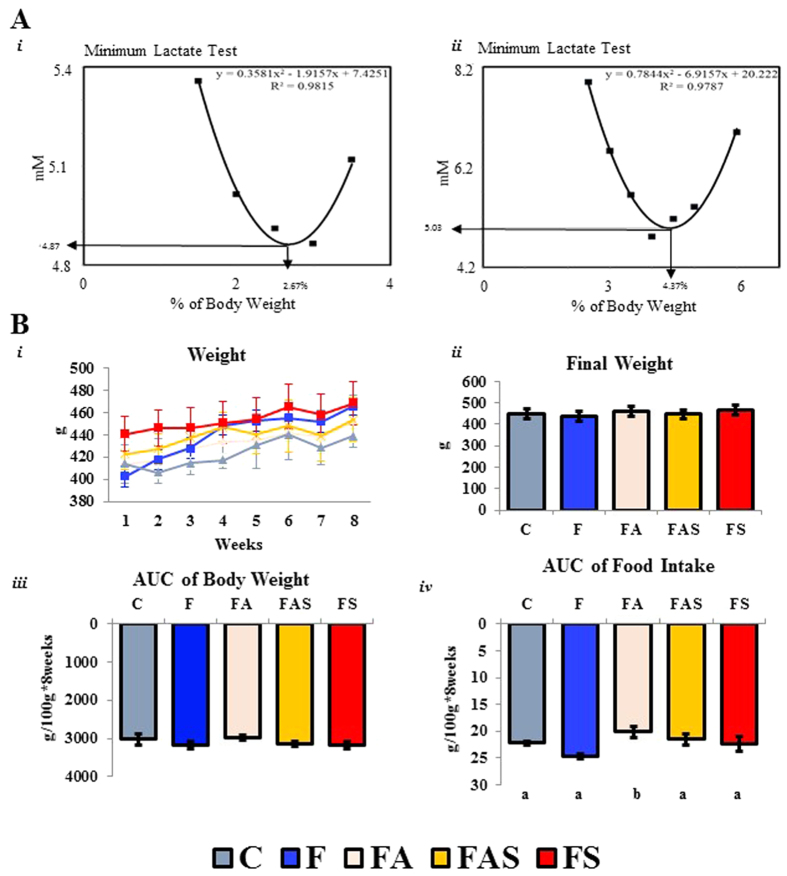
Aerobic exercise seems to reduce food intake and body weight. (**A**) (*i*) Minimum lactate (ML) test in one animal: the estimated ML was 2.67% of body weight, while the interpolated blood lactate concentration was 4.87 mM. In the second animal (*ii*) the estimated ML was 4.37% of body weight, while the interpolated blood lactate concentration was 5.03 mM. (**B**) All of the (*i*) Body weight change, (*ii*) Final Weight, and (*iii*) Area Under Curve of Body Weight did not change during the experiment. (*iv*) F animals showed higher food intake (AUC) than the FA group (p ≤ 0.01). C: Control; F: Fructose; FA: Fructose Aerobic; FAS: Fructose Combined; FS: Fructose Strength. n = 14 animals per group. Different letters mean significant difference.

Figure 1(A) shows the minimum lactate (ML) test in two rats as an example. For the first animal (**1A*****i***), the ML was obtained at a workload of 2.67% of body weight and the blood lactate concentration was 4.87 mM. In the second case (1A***ii***), the estimated ML was obtained at a workload of 4.37% and the blood lactate concentration was 5.03 mM. The results provided by this test were used as standards for each of the two animals during each session of physical exercise and adjusted weekly according to changes in body weight.

### Aerobic exercise reduced food intake but not body weight

Figure 1(B) shows the body weight during the experiment **(1B*****i***), the final weight **(1B*****ii***), the area under curve of body weight **(1B*****iii***), and the AUC of food intake **(1B*****iv***). Figure S1 shows no difference in food consumption week by week. No differences were observed in weight gain and in the AUC of body weight at the end of the experiment. However, the animals in the aerobic (FA) group showed a slight reduction in the AUC of food intake when compared to the Fructose (F) group (p ≤ 0.01). Therefore, aerobic exercise seems to be a good control for the food intake when compared to the other exercise protocols.

### Hyperinsulinemia, glucose intolerance, and insulin resistance were attenuated by aerobic physical exercise, while strength training was able to restore all these parameters to control levels

**Figure 2:**
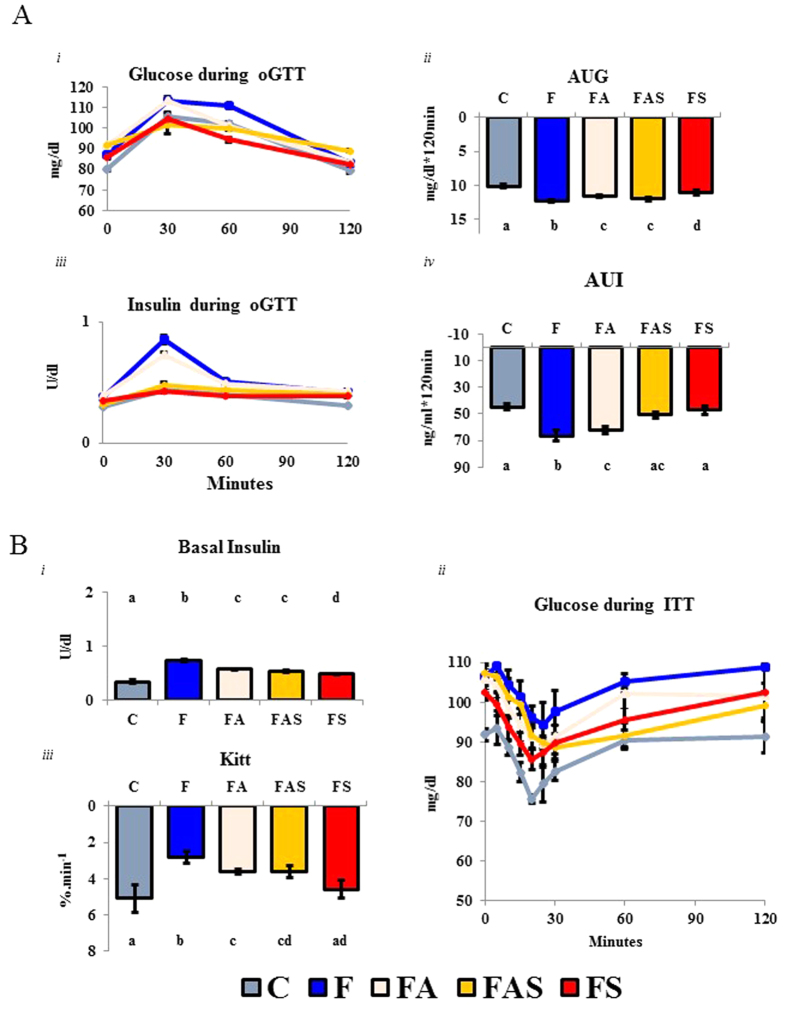
Hyperinsulinemia, glucose intolerance and insulin resistance were attenuated by aerobic physical exercise while strength training was able to restore all these markers to control levels. (**A**) (*i*) Serum glucose kinetics (mg/dl), (*ii*) Area Under Glucose Curve (mg*120 min/dl, AUC) (*iii*) Serum Insulin Kinetics (ng/dl), and (*vi*) Area Under Insulin Curve (ng*120 min/dl, AUC) during the oral glucose tolerance test (oGTT). Animals in the F and FA groups showed higher AUC of glucose than C (p ≤ 0.01) and FS (p ≤ 0.01 *vs* F, and p ≤ 0.05 *vs* FA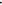 The F group animals showed higher insulin values at 30-minute peak; p ≤ p ≤ 0.01–0.005) than all other groups. In addition, the animals of the F group showed a higher AUC of insulin (p ≤ 0.001–0.005) than all other groups. (**B**) (*i*) The fructose-rich diet was able to induce a hyperinsulinemia (*F diet vs C diet*; p ≤ 0.0001). On the other hand, all exercise protocols were successful in reducing the insulin levels (*Sedentary vs Exercised*; p ≤ 0.01–0.005), but still, these levels were higher than the control animals (p ≤ 0.05–0.01). (*ii*) Glucose kinetics (mg/dl), and (*iii*) Glucose Removal Rate (KITT in %/min^−1^) during the insulin tolerance test (ITT). The F group showed a lower insulin sensitivity (KITT) than all other groups (p ≤ p ≤ 0.001-0.0001). Also, the FA group showed a lower insulin sensitivity than C (p ≤ 0.001) and FS p ≤ −0.005). C: Control; F: Fructose; FA: Fructose Aerobic; FAS: Fructose Combined; FS: Fructose Strength. n = 14 animals per group. Different letters mean significant difference.

There were no differences in serum glucose during the oral glucose tolerance (oGTT) **(2A*****i***). However, during the oGTT, the area under glucose curve (AUG) for the animals in the strength (FS) group **(2A*****ii***) was lower than that in the fructose (F) group (p ≤ 0.05; Fig. 2A). Further, the fructose (F) group had the highest insulin levels among all the other groups at 30 minutes **(2A*****iii***) (p ≤ 0.005) and higher insulin levels than the control group at 120 minutes (p ≤ 0.01) **(2A*****iii***). The fructose (F) group also showed the highest values of area under insulin curve (AUI) among all the other groups (p ≤ 0.005), and more specifically, higher AUI values than the control (C) and strength (FS) groups (p ≤ 0.05) **(2A*****iv***).

Basal Insulin levels were measured using the first sample of the insulin tolerance test (Fig. 2B). All animals fed the fructose-rich diet (F, FA, FAS, and FS) showed higher insulin levels than the control animals (control diet *vs.* fructose-fed diet; p ≤ 0.0001) **(2B*****i***); nevertheless, the animals trained in all exercise protocols had lower insulin levels than the fructose (F) group (F *vs*. FA, FAS, and FS; p ≤ 0.005) ***(i***). Glucose kinetics (mg/dl) and glucose removal rate (kITT in %.min^−1^) during the insulin tolerance test (ITT) are shown in Fig. 2**B*****ii***,***iii***. While fructose (F) group presented lower Kitt values than all of the trained animals (F *vs*. FA, FAS, and FS; p ≤ 0.001) (**2B*****iii***), the strength (FS) animals presented virtually the same levels of insulin sensitivity as the control (C) animals. Taking these results together, while fructose diet promoted insulin resistance (Fructose diet vs Control diet, p ≤ 0.05), strength training was able to return insulin sensitivity to normal values.

### Hypertriglyceridemia was a major feature in rats fed a fructose-rich diet

No differences were observed in serum glucose and total-cholesterol concentrations at the end of the 8-week experiment (Table 1). The fructose diet was able to induce hypertriglyceridemia in all fructose-fed animals (control diet *vs*. fructose-fed diets; p ≤ 0.001). In contrast, the aerobic (FA) and combined (FAS) groups showed higher concentrations of HDL-cholesterol than the control (C) and fructose (F) groups (p ≤ 0.05).

### Physical exercise resulted in an almost complete restoration of the liver microscopic aspect as compared to control animals with a reduction in liver triglycerides and inflammation

Table 2 shows the triglyceride concentration in the liver, heart, soleus muscle, and adipose tissue. The fructose-rich diet-induced triglyceride accumulation in the liver (control diet *vs.* fructose-fed diets; p ≤ 0.0001), and soleus muscle (control *vs*. fructose-fed diets; p ≤ 0.005). However, exercise was able to reduce triglyceride infiltration to lower levels (sedentary *vs*. exercised; liver: p ≤ 0.005; soleus muscle: p ≤ 0.05). Taken individually, the fructose (F) and aerobic (FA) groups had higher concentrations of liver triglycerides than the other groups (p ≤ 0.0001–0.05), followed by the combined (FAS) and strength (FS) groups, which had higher concentrations of liver triglycerides than the control (C) group (p ≤ 0.05). The fructose (F) group also showed an increase in the retroperitoneal triglycerides content as compared to the control (C) and the strength (FS) groups (p ≤ 0.01). All exercised animals showed a higher concentration of soleus muscle triglycerides than the C group (p ≤ 0.05); however, when compared to the F group, all groups had lower concentrations (p ≤ 0.001). No differences were found in the heart and the mesenteric adipose tissue.

**Figure 3:**
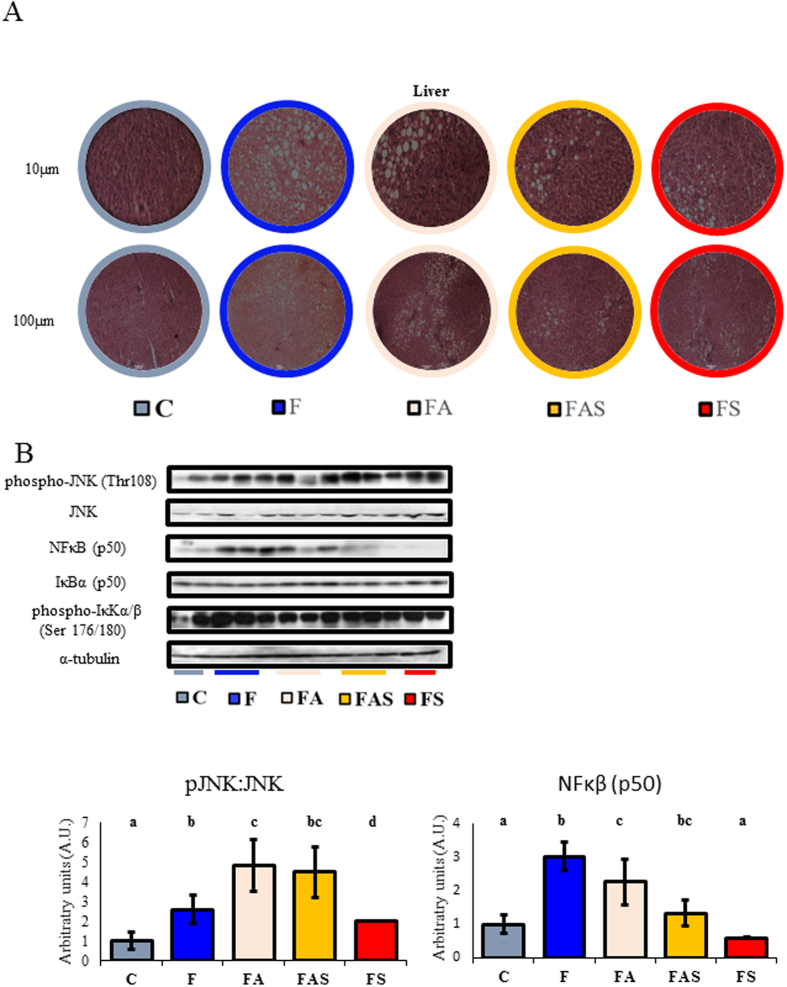
Physical exercise results in an almost complete restoration of the liver microscopic aspect to that of control animals with a reduction in liver triglycerides and inflammation. (**A**) C: Control; F: Fructose; FA: Fructose Aerobic; FAS: Fructose Combined; FS: Fructose Strength. A portion of the right lobe was extirpated to the H&E staining histology. The histology revealed that fructose-fed animals had larger triglyceride storages characterizing diffuse macro vesicular steatosis. (**B**) IκB-α and p-IκKα/β levels were the same for all groups. On the other hand, JNK phosphorylation and total NF-κB (p50) were higher in all Fructose-fed animals than in C (C diet vs F diet, p ≤ 0.01) and in the FA and FAS than in F and FS groups (p ≤ 0.01). Gels have been run at same conditions. For IκB-α, NF-κB (p50), phospho-JNK and JNK two different gels were blotted in the same PVDF membrane to avoid differences in the transfer and exposure. Western blotting signal was detected by special films and later scanned for density quantification. C: Control; F: Fructose; FA: Fructose Aerobic; FAS: Fructose Combined; FS: Fructose Strength. n = 4–6 animals per group. Different letters mean significant difference.

A portion of the right lobe was removed for the hematoxylin and eosin staining histology. The histology revealed that fructose-fed animals had greater levels of storage of triglyceride, which is a characteristic of diffuse macrovesicular steatosis (Fig. 3A).

In the liver, the total inhibitor of nuclear factor kappa B subunit α (IκB-α) and the inhibitor of nuclear factor kappa B kinase subunit α/β phosphorylation (pIKKα/β) were the same for all groups (Fig. 3B). The content of factor nuclear kappa B (NF-κB p50) was increased in F, FA and FAS groups as compared to C; yet, the effect of physical training was remarkable (Sedentary *vs* Exercised p ≤ 0.05). More specifically, strength training (FS) reduced the content of NF-κB p50 to the control levels. On the other hand, pJNK: JNK ratio (phosphorylated c-Jun NH2 terminal kinase: c-Jun NH2 terminal kinase) was increased in all fructose-fed animals (F, FA, FAS and FS) compared to C animals (control diet *vs.* fructose-fed diet, p < 0.01); but again, the strength trained animals (FS) showed a sharper response than the other trained animals.

### Physical exercise attenuates systemic and muscle inflammation

The inflammatory response to the treatment was measured through serum cytokine concentration (Table 3). The fructose (F) animals showed a high-level inflammatory state (Interleukine-4, Interleukine-6, Interferon-γ, and TNF-α) as compared to all other groups (p ≤ 0.01). Serum interleukine-6 (IL-6) concentration was higher in the combined (FAS) group than the C and aerobic (FA) groups (p ≤ 0.0005). All exercise protocols were effective in preventing an increase in some of the inflammatory markers, which are correlated with insulin resistance, obesity, nonalcoholic fatty liver disease, atherosclerosis, and other diseases.

**Figure 4:**
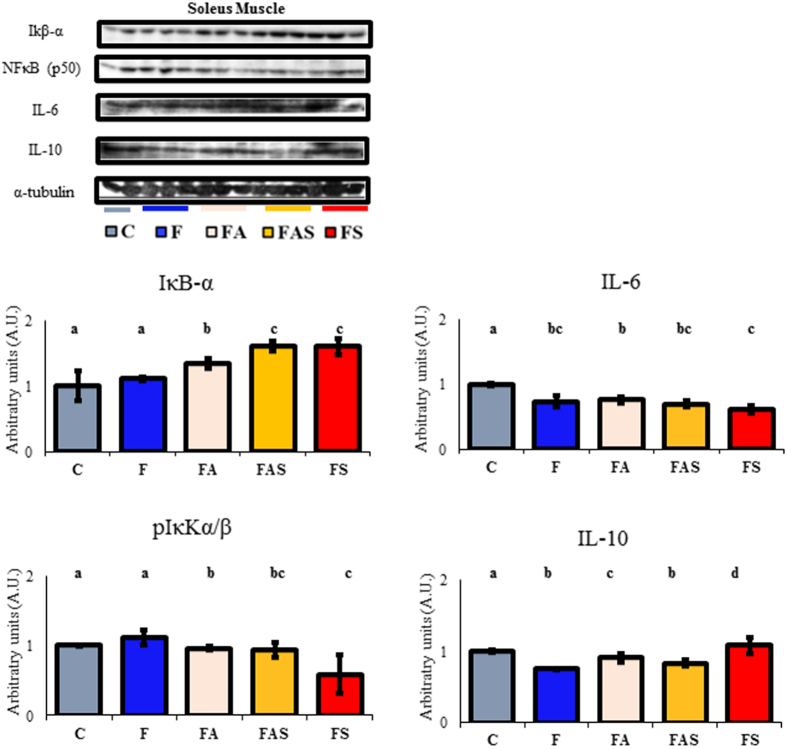
Physical exercise attenuated skeletal muscle inflammation. All exercises protocols increased IKB-α and the aerobic and strength increased IL-10 level in the soleus muscle more than in F (p ≤ 0.05). Also, fructose-exercised animals (FA, FAS and FS) present reduced levels of NFκB (p50) as compared to F (p ≤ 0.05). Gels have been run at same conditions. The western blotting signal was detected by special films and later scanned for density quantification. C: Control; F: Fructose; FA: Fructose Aerobic; FAS: Fructose Combined; FS: Fructose Strength. n = 2–3 animals per group. Different letters mean significant difference.

Extracts from the soleus muscle showed a reduction in the inhibitor of nuclear factor kappa B kinase (IκKα/β) phosphorylation and, consequently, an increase in the inhibitor of nuclear factor kappa B subunit α (IκB-α) content, with no changes in the NF-κB (p50) expression (Fig. 4). When assessing the c-Jun-NH2-terminal Kinase (JNK), we found no difference in phosphorylation or total JNK (data not shown). Also, the fructose diet was able to reduce IL-10 expression (C diet *vs.* F diet; p ≤ 0.05). Better increments were found in the strength (FS) group than the combined (FAS) (p ≤ 0.02) or the aerobic (FA) (p ≤ 0.002) groups. On the other hand, the fructose-fed diets reduced IL-6 as compared to the control diet (C diet *vs.* F diets; p ≤ 0.0001) and exercise was not able to improve these levels.

**Figure 5:**
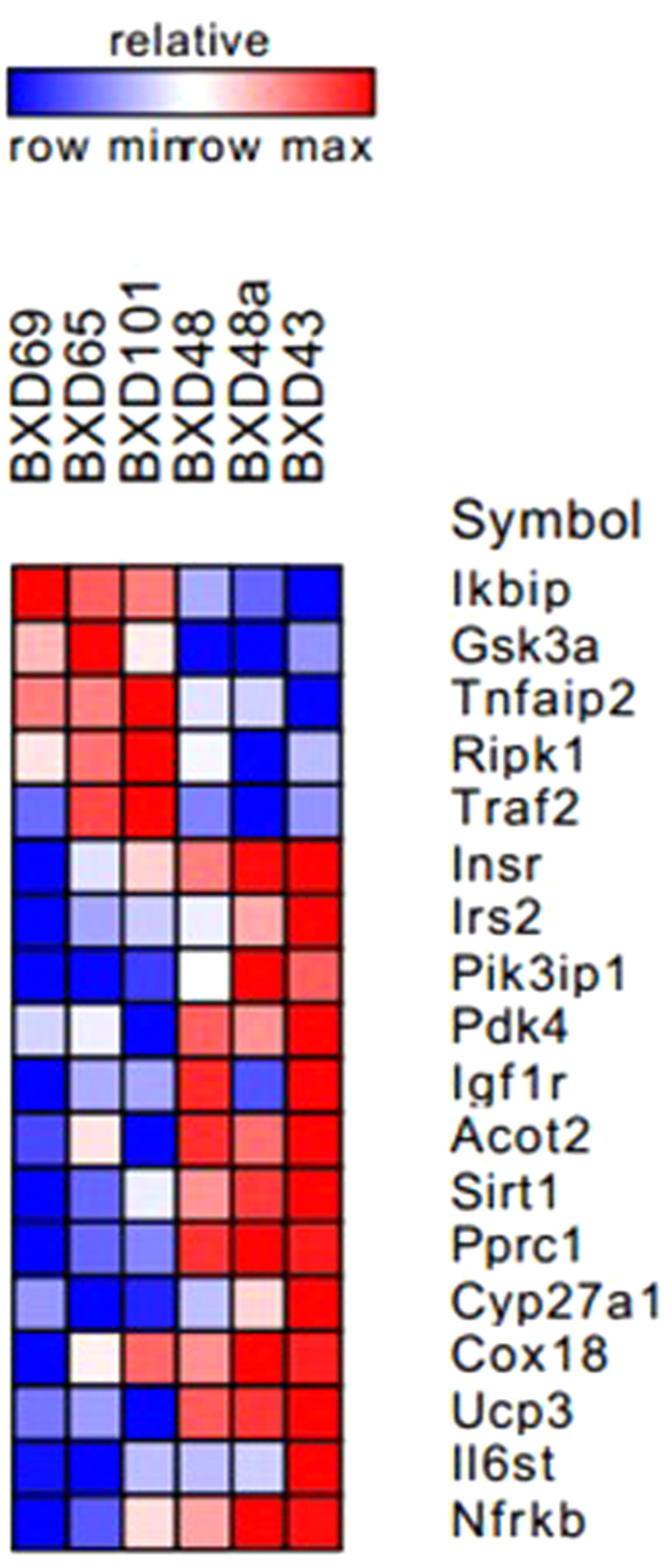
Heatmap correlating IKbip genes to insulin pathway, inflammatory cytokines, and mitochondrial biogenesis markers. The heatmap contains data from mRNA obtained from a large cohort of BXD family (mice strains generated by crossing C57BL/6J and DBA/2J); *(row max is related to higher levels of each gene, in each row, being red for the positive correlation and blue negative correlation*). Gene functions/pathways: IKBIP: chosen inflammatory marker; GSK3A: gluconeogenesis marker; TNFAIP, RIPK1, TRAF2: inflammatory markers; INSR, IRS2, PIK3IP1, PDK4, IGF1R: genes related to insulin pathway; ACOT2, SIRT1, PPRC1, CYP27A1, COX18: genes related to mitochondrial functions; UCP3: gene related to energy expenditure; IL6st: gene related to exercise-induced adaptation; NFRKB: gene inversely related to IKBIP levels.

Using BXD murine data set for multiscalar integration of trails^20^, we then evaluated possible correlations between gene expression of the IKbip, genes involved in the insulin pathway, and mitochondrial genes, which are expected to be affected by the training regimen. The results were summarized in a heatmap (Fig. 5), showing a close relationship between inflammatory genes and the IKbi, which were inversely correlated to genes representing adaptations from physical training.

## Discussion

In this 8-week pre-clinical experiment, we found that the hyperinsulinemic and inflammation responses to the fructose-rich diet administration were attenuated, or, in some cases, even prevented by all exercise protocols. More specifically, the improvements seen in the glucose tolerance and insulin sensitivity tests seemed to be more responsive to the strength exercise protocol than the aerobic or combined protocols.

Aerobic exercise has been effective in reducing food intake in animals fed a fructose-rich diet. While this association has been previously described in humans^21^, our data corroborate animal study delineated by Ropelle and colleagues^22^ where, endurance physical exercise prevented inflammation in the ventromedial hypothalamus, which in part, could ameliorate the hunger mechanism. On the other hand, we did not find any reduction or increase in hunger in animals in the combined (FAS) or strength (FS) protocol; this led us to believe that at least in eutrophic mice, these protocols may not be the best strategy to reduce food intake.

The individual effects of the three different exercise protocols became more pronounced when assessing the oral glucose tolerance test, insulin, and insulin sensitivity. Strength exercise seemed to have greater effects on the regulation of these parameters; a “progressive effect” was seen where the response was the lowest in the aerobic (FA) group, intermediate in the combined (FAS) group, and the highest in the strength (FS) group. Similar results were seen in humans^23^. It is worth mentioning that the values of the strength (FS) group were similar to those in the control group for most of these results.

Hyperinsulinemia is perhaps the most important trigger for all metabolic disorders^24^. An excess of this hormone appears to block energy expenditure in animals and to activate the “economy mode” in the body while increasing the dependence on lipids, which would result in an inflammatory process in several tissues^25^,^26^. All exercise protocols were effective in reducing the concentrations of circulating insulin, corroborating studies in humans^27^,^28^ and animal models^29^. On the other hand, strength training provided sharper responses as compared to all other fructose- fed animals. We attribute this finding to the ability of this specific training to reduce inflammation and liver lipid content in our animals, which are both correlated to insulin resistance and hyperinsulinemia^30^,^31^,^32^.

As for the serum parameters (glucose, triglycerides, HDL-cholesterol, and total cholesterol), exercise exerted only a small change. HDL-cholesterol in the aerobic (FA) and combined (FAS) groups showed a considerable increase as compared to the control, fructose (F), and strength (FS) groups. The cholesterol pathway was not evaluated in this study, but the improvement in liver function or the increased consumption of triglycerides due to aerobic exercise may have caused these changes. The workload that was used and standardized in our study (80% of Minimum lactate) corresponds to the transition between moderate and vigorous exercise intensity^33^. At this particular point, both carbohydrates and lipids contribute virtually equally to the energy demand^34^. This effect could be responsible for any improvement in the lipid metabolism of the animals, thus, improving the concentrations of HDL-cholesterol and corroborating previous studies in animals^15^,^35^,^36^. On the other hand, a fructose-rich diet increased serum triglyceride concentration by almost 65%. This accumulation may have been triggered by denovo lipogenesis as described by Basciano^37^ and Loria^38^.

Lipid accumulation in the liver and bloodstream became more pronounced when rats were fed a fructose-rich diet. Exercise was able to reduce this hepatic lipid accumulation, in addition to its effects on the circulating triglyceride concentration. Notably, disruption in the liver lipid content can be triggered by fructose via denovo liponeogenesis^37^; this excess can spill over into the bloodstream and can infiltrate the heart, soleus muscle, and retroperitoneal adipose tissue of the animals. Concordant with previous studies in animals^39^ and humans^40^, the three exercise protocols were successful in reducing the concentration of hepatic triglyceride; but, surprisingly, the strength (FS) group had the smallest reserves of hepatic triglyceride and the lowest concentrations of circulating insulin among all the exercised groups. Once again, we can observe a “progressive effect” among the four groups fed a fructose-rich diet (F, FA, FAS, and FS) similar to the complete restoration of the liver microscopic aspect. This is congruent with the studies by Mehran and colleagues^24^ that showed that a reduced concentration of circulating insulin in Ins1^+/−^; Ins2^−/−^ knockout mice triggered a protective effect when fed a high-fat diet.

We also analyzed the levels of inflammatory markers (cytokines) in all of the groups. Exercise favorably attenuated the serum concentrations of four (IL-4, IL-6, interferon-γ, and TNF-α) of the six assessed cytokines. Interleukin-6 (IL-6) has an ambiguous role; it could play a pro- or an anti-inflammatory role depending on the target tissue. In the absence of exercise, high blood levels of this cytokine can generate a supra-physiological inflammatory response, which interferes in several key processes in the regulation of glucose homeostasis and could contribute to insulin resistance^41^; however, after a strenuous exercise, serum IL-6 concentration increases in the muscle by 100-fold up to 24 hours due to muscle contractions^42^; consequently, this cytokine increases insulin sensitivity, improves glucose tolerance^43^, and plays a key role in rebuilding muscle cells and fighting infections through the activation of macrophages^40^,^44^. In the present study, animals fed a fructose-rich diet presented a high circulating concentration of IL-6. Our data was successful in achieving both systemic and muscular levels of IL-6. As expected, physical exercise was responsible for the great reductions in systemic IL-6 levels; yet, no difference was found in the muscle of all fructose-fed and trained animals even sixteen hours after the last exercise session; this led us to assume that a possible acute IL-6 release in the muscle, 3–4 hours after the exercise, might have been responsible for downregulating the chronic serum release of this cytokine and its deleterious effects. Moreover, IL-4, TNF-α, and interferon-γ are exclusively inflammatory cytokines^45^,^46^,^47^; these were found to be elevated in the fructose sedentary group, while the exercised animals had a lower concentration of all these three cytokines levels. These three molecules can affect the insulin signaling cascade by preventing the normal functioning of the insulin receptor and all kinases involved in this reaction chain.

As for the protein analysis in the soleus muscle, all three exercise protocols were able to reduce the inflammatory pathways. NFκB/IκB-α is one of the most powerful triggers to activate inflammation and promote cytokine release^48^. Our data suggest that improvements in the soleus muscle due to chronic exercise led to a protective effect against inflammation and its associated insulin resistance. Even if muscular IL-6 levels were found to be reduced in all fructose-fed animals, the release of this cytokine after exercise is transient and longer responses can be found in the IL-10 expression^22^,^46^. IL-10 expression is increased in both FA and FS groups compared to fructose-fed sedentary animals. According to Ropelle e colleagues^22^, IL-6 is responsible for IL-10 expression which mediates the suppression the NFκB pathway via IKKαβ inhibition^49^,^50^. On the other hand, no differences in JNK phosphorylation were found in the liver (data not shown) of any animal. In the liver, physical exercise seemed to provide a better outcome in reducing the NFκB content associated with a non-expected increase in JNK phosphorylation in both aerobic and combined exercises than all the other groups. Strength exercise, however, led to a reduced JNK phosphorylation as compared to all the fructose-fed groups and to a smaller increase than the control animals.

To explore the potential influence of IKKαβ levels among various processes related to exercise adaptation in the skeletal muscle, we performed a bioinformatics analysis using BXD murine data set for multiscalar integration of traits, as previously published^20^. In this analysis we found information supporting our rationale that *IKbip* gene expression was inversely correlated to insulin pathway genes and positively correlated to other inflammatory genes. Our data showed that strength training provided the most robust reduction of IKKαβ phosphorylation, which in turn led to a reduced inflammation in the animals’ muscle.

In conclusion, although all exercise modalities improved metabolic health in rodents, our data demonstrated for the first time that strength training was the most effective in preventing detriments in markers of metabolic health in rodents fed a fructose-rich diet. This preclinical experiment advances our understanding of how different exercise modalities alter molecular pathways linked to glucose tolerance, insulin sensitivity and lipid handling in the liver and the skeletal muscle, regardless of body weight changes. This study identified some of the molecular transducers of exercise that improved health and set the stage for the establishment of exercise guidelines to prevent fructose-induced metabolic derangements. Not only do these results build up a new basis for future studies in humans, but they also open up new possibilities for the exercise to be used as a polyvalent tool in studies aiming to alter body weight, feeding behavior, and/or caloric expenditure.

## Materials and Methods

Male Wistar rats (*Rattus Norvegicus;* 120 days old, n = 70) were housed in laboratory cages (four animals per cage) at a controlled temperature of 25 °C ± 1 °C and under a 12/12-hour light/dark cycle with free access to water and food. The experiment was performed at the Nutrition, Metabolism and Exercise Laboratory at São Paulo State University, Rio Claro, Brazil. All experiments were analyzed and approved by the Biosciences Institute Animal Ethics Committee, Rio Claro Campus (case number: 005/2010) and were carried out in accordance with the approved guidelines.

### Diet composition

Laboratory Commercial rodent chow (Labina^®^, Purina) was used as a control diet (57.3% carbohydrate, 41.2% cornstarch), as this diet has been used in previous studies as a control diet^51^,^52^.

For the fructose-rich diet, we used an adapted diet that was standardized by Bezerra and colleagues^53^ composed of 202 g/kg casein, 625.5 g/kg fructose, 2 g/kg l-cysteine, 70 g/kg soy oil, 35 g/kg mineral salt mix, 10 g/kg vitamin mix^54^, 50 g/kg fiber, and 2.5 g/kg choline chloridrate. This diet was used for its characteristics of triggering metabolic disorders independent of weight changes^19^.

### Experimental groups

At 120 days old, the animals were randomly separated into five groups: **1**) The Control Group (C) was fed the commercial balanced diet from 120 to 180 days old; **2)** The Fructose Group (F) was fed the semi-purified fructose-rich diet from 120 to 180 days old; **3)** The Fructose Aerobic Group (FA) fed the semi-purified fructose-rich diet from 120 to 180 days old, and these animals were trained during the same period as per the aerobic exercise protocol for one hour per day, five days per week; **4)** The Fructose Combined Group (FAS) was fed the semi-purified fructose-rich diet from 120 to 180 days old, and these animals were trained during the same period as per the aerobic and strength exercise protocols on alternate days, five days per week; **5)** The Fructose Strength Group (FS) was fed the semi-purified fructose-rich diet from 120 to 180 days old, and these animals were trained as per the strength exercise protocol five days per week; (n = …). The experimental design is provided in supplementary Figure S1.

### Physical training

As postulated by the theory of training and exercise physiology, strength training and aerobic training (endurance) are two different entities and they must be applied differently^55^. Supported by this fact, we randomized rats to three different exercise protocols independent of calorie expenditure.

#### Aerobic protocol

According to previous research from our laboratory^56^, Wistar rats swam in individual tanks of water (75 cm deep) at 31 °C ± 1 °C for one hour per day, five days per week. Exercise was performed at 80% of the individual minimum lactate intensity overload attached to the animal thorax^35^.

#### Strength protocol

According to previous research from our laboratory^57^, animals performed jumps in individual tanks of water (75 cm deep) temperature at 31 °C ± 1 °C. Animals performed four series of 10 jumps with a 50% body weight overload attached to the thorax and a one-minute rest between series for five days per week^35^.

#### Combined protocol

Animals were trained with the aerobic protocol three times a week (Mondays, Wednesdays, and Fridays) and with the strength protocol twice a week (Tuesdays and Thursdays).

### Body weight

Body weight was recorded weekly during the experimental phase using a semi-analytic scale (Model S5201, Bel Company, Brazil), and the area under the curve (AUC) was calculated with Microsoft Excel 2013 software using the trapezoidal method^58^.

### Food intake

Food intake was recorded once a week during the experimental phase, the values were than divided by the weight of animals and relativized to g/100 g. The AUC was calculated using Microsoft Excel 2013 software through the trapezoidal method^58^.

### Oral glucose tolerance test

The oral glucose tolerance test (oGTT) was performed in animals after a 12-hour fast and 16 hours after the last exercise bout. First, a blood sample was collected from the tail (fasting), and then, a 20% glucose solution (2 g/kg body weight) was administered to the rats by a polyethylene gastric tube. Blood samples were collected after 30, 60, and 120 minutes into heparinized capillary tubes calibrated for 25 μL to establish the glucose and insulin concentrations. The blood glucose concentration was measured using the glucose-oxidase method^59^ and the serum insulin levels were measured using an ultrasensitive mouse insulin ELISA kit (EZRMI-13 K.EIA; Millipore, St. Charles, MI). Results were analyzed by establishing the serum glucose AUG values by means of the trapezoidal rule using Excel 2013^58^.

### Insulin tolerance test

Insulin sensitivity was assessed 16 hours from the exercise bout by means of a subcutaneous insulin tolerance test (ITT), which consisted of subcutaneous administration of Humalog^®^ insulin (300 mU/Kg body weight; Lilly^®^, São Paulo, Brazil), followed by blood sampling at 0, 5, 10, 15, 20, 25, 30, 60, and 120 minutes. The blood glucose removal rate (KITT), expressed as %/minute. The t/2 blood glucose was calculated by a least-squares analysis of the curve of serum glucose contents, as long as a linear decrease after insulin administration was evident^60^. We also calculated serum area under glucose curve values by means of the trapezoidal rule using Excel 2013^58^.

### *In vitro* assays

#### Biological material

Animals were sacrificed 48 hours after the last *in vivo* test and 16 hours after the last exercise bout in a CO_2_ chamber after 4 hours of fasting. Blood samples were collected via the liver portal vein for three different assays. One sample was used to measure glucose, triglycerides, HDL-cholesterol, total cholesterol, and basal insulin concentrations by means of a commercial kit (Laborlab^®^, São Paulo, Brazil) and an ELISA kit (EZRMI-13 K.EIA: Millipore, St. Charles, MI). Another sample was used to assess IL-4, IL-1β, IL-6, IL-10, INF-γ, and TNF-α. Samples from different tissues were removed to assess the triglyceride concentrations or snap frozen in liquid nitrogen, and then placed at −80 °C for future Western blotting analysis.

#### Liver hematoxylin-eosin histology

Liver samples were collected and fixed in Bouin’s fixative. The tissue was mounted in HistoResin (Leica Embedding Kit) and sliced in a microtome (Leica RM2145) to a thickness of 6 μm. The slices were subjected to the hematoxylin-eosin (H&E) staining method. The slices were hydrated and stained with hematoxylin (10 minutes), and were then washed and stained with eosin (5 minutes). Finally, they were washed and preserved in a Canadian balsam.

#### Western blot analysis

Samples of soleus muscle were quickly removed 16 hours after the last training bout, washed with phosphate buffer solution, minced coarsely, and homogenized in freshly prepared radioimmuno precipitation assay buffer (10 mM Tris-HCl, 1 mM ethylene diamine tetraacetic acid (EDTA), 0.5 mM ethylene glycol tetraacetic acid (EGTA), 1% Triton-X, 0.1% sodium deoxycholate, 0.1% sodium dodecyl sulfate (SDS), 140 mM sodium chloride (NaCl), 2 μg/ml aprotinin, 1 mM phenylmethanesulfonyl fluoride (PMSF), 1 mM sodium orthovanadate. 5 mM sodium fluoride which is suitable for preserving phosphorylation and integrity of the protein extract. Western blotting was performed as described in previous research^24^.

#### Antibodies and materials

Proteins were separated using polyacrylamide gel and then transferred to polyvinilidene fluoride (PVDF) membranes (GVS^®^) and stained in Ponceau solution, followed by antibodies incubation using: anti-IκB-α (rabbit, Cell Signaling^®^), anti-phospho-IκKα/β (Ser 176/180) (rabbit, Cell Signaling^®^ ), anti-NFκB p65/50 (rabbit, Cell Signaling^®^ ), anti-phospho-JNK (Thr138/135) (rabbit, Cell Signaling^®^ ), anti-JNK (rabbit, Cell Signaling^®^ ), anti-IL6 (goat, Santa Cruz^®^ ), anti-IL10 (goat, Santa Cruz^®^ ), and anti-α-tubulin (mouse, Novus Biological^®^ ).

### Bioinformatics Analysis

The GeneNetwork (www.genenetwork.org) was used for all genetic analyses. Pearson’s *r* and p values were calculated to determine magnitude and significance of correlations except when otherwise indicated. We than, used the gene-e software to create a heatmap figure.

### Statistics

The Shapiro-Wilk’s W test was used to verify the normality of the sample. Results were analyzed using a two-way ANOVA and, when necessary, a *post-hoc* Bonferroni test was performed. The significance level was set at 0.05. Statistica 7.0^®^ software was used for all analyses.

## Additional Information

**How to cite this article**: Botezelli, J. D. *et al*. Strength Training Prevents Hyperinsulinemia, Insulin Resistance, and Inflammation Independent of Weight Loss in Fructose-Fed Animals. *Sci. Rep.* **6**, 31106; doi: 10.1038/srep31106 (2016).

## Supplementary Material

Supplementary Information

## Figures and Tables

**Table 1:** Glucose, Triglycerides, HDL cholesterol, and Total-cholesterol concentrations, in the animals’ serum at the end of the experiment.

Groups	Glucose (mg/dL)	Triglycerides (mg/dL)	HDL-Cholesterol (mg/dL)	Total-Cholesterol (mg/dL)
C	98.8 ± 3.50	172.2 ± 15.02^a^	22.7 ± 1.20^a^	78.9 ± 5.58
F	110.9 ± 5.02	261.4 ± 4.49^b^	22.3 ± 1.48^a^	89.1 ± 5.44
FA	106.4 ± 3.35	262.4 ± 9.05^b^	31.3 ± 1.13^b^	85.9 ± 1.69
FAS	105.0 ± 5.19	235.4 ± 12.65^b^	35.2 ± 2.51^b^	70.7 ± 7.03
FS	104.5 ± 4.98	242.0 ± 7.8^b^	24.4 ± 1.09^a^	79.9 ± 8.23

C: Control; F: Fructose; FA: Fructose Aerobic; FAS: Fructose Combined; FS: Fructose Strength. n = 14 animals per group. Different letters mean significant difference. No differences were observed in the serum Glucose and Total-Cholesterol concentrations at the end of experiment. All fructose-fed animals (F, FA, FAS and FS) had higher concentrations of serum triglycerides as compared to C (p ≤ 0.0001–0.01). Also, FA and FAS group had higher concentrations of HDL-Cholesterol as compared to C and F groups (p ≤ 0.05).

**Table 2:** Triglycerides concentrations (mg/dL) in the liver, heart and adipose tissue (Mesenteric, Retroperitoneal and Subcutaneous regions) at the end of the experiment.

Groups	Liver (umol/mg)	Heart (umol/mg)	Soleus (umol/mg)	Mesenteric (umol/mg)	Retro-Peritoneal (umol/mg)
C	2.7 ± 0.8^a^	0.64 ± 0.07	0.31 ± 0.02^a^	46.1 ± 1.83	70.1 ± 1.94^a^
F	16.5 ± 0.35^b^	0.71 ± 0.03	1.34 ± 0.11^b^	57.5 ± 3.46	115.7 ± 10.11^b^
FA	11.5 ± 0.32^c^	0.56 ± 0.04	0.45 ± 0.03^c^	44.9 ± 4.87	81.7 ± 5.44^c^
FAS	6.8 ± 0.32^d^	0.53 ± 0.03	0.59 ± 0.06^c^	48.1 ± 3.11	93.3 ± 9.01^c^
FS	4.6 ± 0.15^e^	0.55 ± 0.07	0.67 ± 0.04^c^	51.9 ± 4.56	65.7 ± 3.25^a^

C: Control; F: Fructose; FA: Fructose Aerobic; FAS: Fructose Combined; FS: Fructose Strength. n = 14 animals per group. Different letters mean significant difference. F and FA groups had increased liver triglycerides’ concentrations as compared to the other groups (p ≤ 0.0001–0.05), while the FS group had a lower liver triglycerides’ concentration than F, FA, and FAS (p < 0.05); F, FA, FAS, and FS had higher concentrations of triglycerides in the liver than the C group (p ≥ 0.05). The F group had a higher retroperitoneal triglycerides’ content than the C and FS groups (p ≤ 0.001–0.01). All exercised animals had a higher concentration of muscle triglycerides than the C group (p ≤ 0.05) but lower concentrations than F (p ≤ 0.001).

**Table 3:** Cytokines concentration in blood samples of animals fed the fructose-rich diet.

Groups	IL-4 (pg/mL)	IL-1β (pg/mL)	IL-6 (pg/mL)	IL-10 (pg/mL)	Interferon-γ (pg/mL)	TNF-α (pg/mL)
C	2.37 ± 0.41^**a**^	27,75 ± 2.29	4.87 ± 0.52^**a**^	9.87 ± 0.57	10.81 ± 1.03^a^	8.56 ± 0.51^a^
F	5.32 ± 0.79^**b**^	22.85 ± 2.21	52.1 ± 4.54^**b**^	12.07 ± 0.65	80.35 ± 6.28^**b**^	21.9 ± 0.35^**b**^
FA	1.97 ± 0.24^**c**^	21.59 ± 1.68	5.81 ± 0.86^**c**^	10.18 ± 0.59	21.22 ± 3.09^**b**^	8.77 ± 0.50^c^
FAS	2.66 ± 0.31^**c**^	23.16 ± 1.98	18.33 ± 1.86^**d**^	11.03 ± 0.68	23.16 ± 2.72^**b**^	11.04 ± 0.52^**c**^
FS	2.37 ± 0.65^**ac**^	25.50 ± 2.24	11.75 ± 0.77^**e**^	12.43 ± 0.82	14.12 ± 1.87^**a**^	10.18 ± 0.24^**c**^

C: Control; F: Fructose; FA: Fructose Aerobic; FAS: Fructose Combined; FS: Fructose Strength. n = 14 animals per group. Different letters mean significant difference.

The inflammatory response to the treatment shown in Fig. 5 was achieved through the serum cytokines’ concentrations. The F animals showed a high-level of inflammatory state (*ii.* IL-4, *iv.* IL-6, *v*. Interferon-γ and *vi*. TNF-α) as compared to all other groups (p ≤ 0.01–0.001). Serum IL-6 concentration (*iv*) was higher in the FAS group than the C and FA groups (p ≤ 0.0001–0.0005). All exercises protocols were effective in preventing an increase in some inflammatory markers correlated with insulin resistance, obesity, NASH, atherosclerosis and other correlated diseases.
