# Supplementary material for: Strength Training Prevents Hyperinsulinemia, Insulin Resistance, and Inflammation Independent of Weight Loss in Fructose-Fed Animals
Source: Sci Rep. 2016 Aug 4;6:31106. doi: 10.1038/srep31106 (PMC4973231; doi:10.1038/srep31106)
Supplement: Supplementary Information [file srep31106-s1.pdf]

SUPPLEMENTARY INFO

STRENGTH TRAINING PREVENTS HYPERINSULINEMIA, INSULIN RESISTANCE, AND INFLAMMATION INDEPENDENT OF WEIGHT LOSS IN FRUCTOSE-FED ANIMALS

José D. Botezelli [1,2*], Andressa Coope [2], Ana C. Ghezzi [2], Lucieli T. Cambri [3], Leandro P. Moura[1], Pedro P.M. Scariot [1], Rodrigo Stellzer Gaspar[1], Rania A. Mekary [4,5], Eduardo Rochete Ropelle[1], José Rodrigo Pauli [1]

1. Department of Nutrition, Metabolism and Exercise, Campinas State University (UNICAMP) in Limeira/SP-Brazil

2. Medical Sciences University, Campinas State University (UNICAMP) in Campinas/SP-Brazil

3. Department of Physical Education, Mato Grosso Federal University (UFMT) in Cuiabá/MT-Brazil

4. Department of Pharmaceutical Business and Administrative Sciences; MCPHS University, Boston/MA, USA.

5. Department of Surgery; Brigham and Women's Hospital, Harvard Medical School, Boston/MA, USA.

Training Adaptation

To avoid the stress effects of the exercise, the animals were subjected to a short-term adaptation[1] before the experiment (95-110d of age). All animals were subjected to the adaptation.

Aerobic training adaptation

The sedentary (C) and aerobically trained (FA and FAS) groups were first adapted to the water environment. Adaptation was performed over10 uninterrupted days in the same tank where the training was performed. The water temperature was kept at 31 ± 1°C.[2] The aim of adaptation was to reduce animal stress and to avoid possible physiological adaptations that might improve the physical capacity of the animals.

Rats were placed in shallow water for 10 min for three days. The water depth was then increased, as was the effort length and load (1% body weight in the form of lead ballasts placed in a Velcro® backpack attached to the thorax) carried by the animals. By the fourth day, the animals swam for 5 min in deep water (75cm). The length of time was increased by 10min each day until the 12th day of adaptation.[2]

Strength training adaptation

Rats were placed in shallow water tanks (31 ± 1°C) for 10 min the first two days. On the third, fourth, and fifth days, the depth level was increased and the animals were kept in the tanks for 5, 10, and 15 min, consecutively. On the sixth and seventh days, a 30% body weight overload in a Velcro® "backpack" was attached to the thorax of the animals and they were kept into the tank with shallow water. In the last three days, the animals performed 10 jumps with a 30%

overload attached to the thorax, while the depth of water was progressively increased (25, 50 and 75cm).[3]

Combined training adaptation

Rats were placed in shallow water (31 ± 1°C) for 10 min on the first two days. On the third, fourth, and fifth days, the depth level of the water was increased and the animals were kept in the water for 5, 10, and 15 min, consecutively. On the fifth, sixth, and seventh days, the animals were subjected to increased exposure times (5 min per day) in the water with a 1% body weight load attached by means of a Velcro® backpack. On the last three days, the animals were subjected to strength training adaptation: On the first day, they were kept in shallow water with a load attached to the thorax (30% body weight); during the following days, they performed 10 jumps carrying this same load inside tanks filled up to 50% or 100% of their maximum capacity.

Lactate Minimum

The aerobic training was performed using 80% of the lactate threshold; this domain corresponds approximately to 65-70% of the VO_2 Max. The lactate threshold during swimming was calculated by determining the adapted "minimum lactate" test[2]. For this test, 110d old animals were initially placed individually in tanks (100 cm X 80 cm X 80 cm) containing water at 31 ± 1°C. Animals carried an overload that was 13% of their body weight to induce hyperlactacidemia and were then exercised for 30 sec. After resting for 30 sec, they swam carrying the 13% load until exhaustion. After a 9min rest, a blood sample was collected by means of a cut in the distal end of the tail to determine lactate concentration. Then, animals performed exercise with progressively heavier loads[2,5]. The initial load was 2% of the body

weight of the animal; the load was increased 0.5% every 5 min until exhaustion. After each load change, a blood sample was collected to measure lactate. The lactate minimum speed (ML) was determined using a second-order polynomial curve adjusted to the blood lactate vs. workload curve. Blood lactate concentration was measured by spectrophotometry.[4] The lowest lactate concentration on the curve (minimum lactate) theoretically represents the maximum exercise intensity, where lactate production and removal occur in the same proportions.[5]

Inflammatory Markers

The blood samples were collected via portal-vein puncture from a minimum of eight animals from each group. Samples were centrifuged at 6,000 g for 10 min, filtered through a 1.2-mm polyvinylidene fluoride filter plate (Millipore, Billerica, MA, USA), and then incubated overnight using a Milliplex$_{MAP}$® Rat Cytokine/Chemokine Immunoassay. A sample of 25μl was used to analyze all cytokines (IL1-β, IL-4, IL-6, IL-10, INF-γ and TNF-α). Cytokines were quantified using the equipment Luminex^{200} (Luminex Corporation, Austin, TX, USA) and the software xPonent 3.1®. MILLIPLEX™ MAP is based on the Luminex® xMAP® technology that uses a technique to internally color-code microspheres with two fluorescent dyes. Through precise concentrations of these dyes, 100 distinctly colored bead sets can be created, each of which is coated with a specific capture antibody. After an analytic from a test sample is captured by the bead, a biotinylated detection antibody is introduced in the reaction mixture, which is then incubated with Streptavidin-Phycoerythrin conjugate, the reporter molecule, to complete the reaction on the surface of each microsphere. The microspheres are allowed to pass rapidly through a laser which excites the internal dyes marking the microsphere set. A second laser excites Streptavidin-Phycoerythrin, the fluorescent dye on the reporter molecule. Finally, high-

speed digital-signal processors identify each individual microsphere and quantify the result of its bioassay based on fluorescent reporter signals.

References

1. Botezelli, J. D. *et al.* Different exercise protocols improve metabolic syndrome markers, tissue triglycerides content and antioxidant status in rats. *Diabetol. Metab. Syndr.* **3,** 35 (2011).
2. Gobatto, C. A. *et al.* Maximal lactate steady state in rats submitted to swimming exercise. *Comp. Biochem. Physiol. A. Mol. Integr. Physiol.* **130,** 21–7 (2001).
3. Rogatto, G. P. Influência do Treinamento Físico Intenso Sobre o Metabolismo de Proteínas. **7,** 75–82 (2001).
4. Engel, P. C. & Jones, J. B. Causes and elimination of erratic blanks in enzymatic metabolite assays involving the use of NAD+ in alkaline hydrazine buffers: improved conditions for the assay of L-glutamate, L-lactate, and other metabolites. *Anal. Biochem.* **88,** 475–84 (1978).
5. Voltarelli, F. A., Gobatto, C. A. & de Mello, M. A. R. Determination of anaerobic threshold in rats using the lactate minimum test. *Braz. J. Med. Biol. Res.* **35,** 1389–94 (2002).

Supplementary Figures

Supplementary Figure 1. Exercise does not change linear food intake after eight weeks experiment.

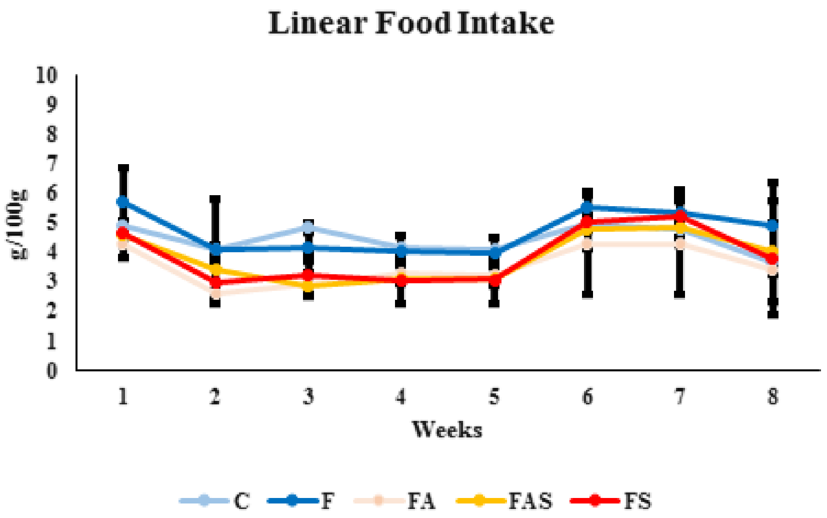

C: Control; F: Fructose; FA: Fructose Aerobic; FAS: Fructose Combined; FS: Fructose Strength. n = 14 animals per group.

Supplementary Figure 2. Liver Western Blotting Results.

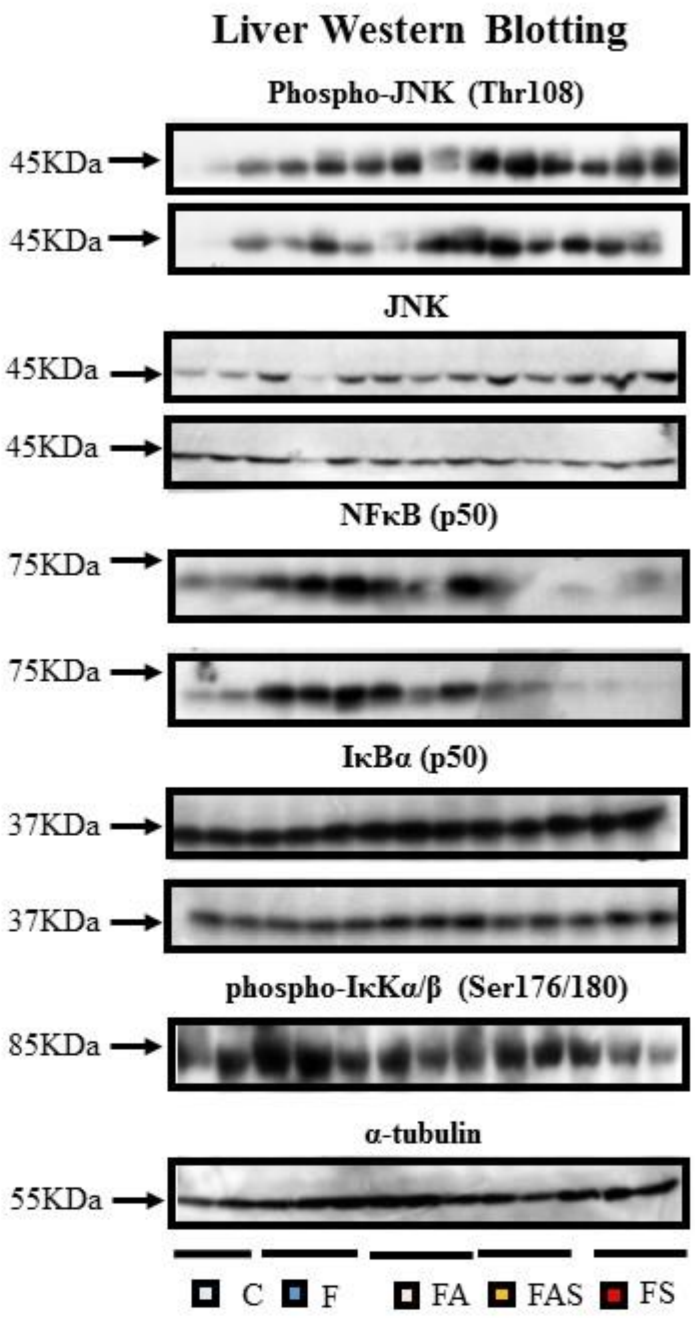

Gels have been run at same conditions. For IκB-α, NF-κB (p50), phospho-JNK and JNK two different gels were blotted in the same PVDF membrane to avoid differences in the transfer and exposure. Western blotting signal was detected by special films and later scanned for density

quantification. C: Control; F: Fructose; FA: Fructose Aerobic; FAS: Fructose Combined; FS: Fructose Strength.

Supplementary Figure 3. Soleus Muscle Western Blotting Results.

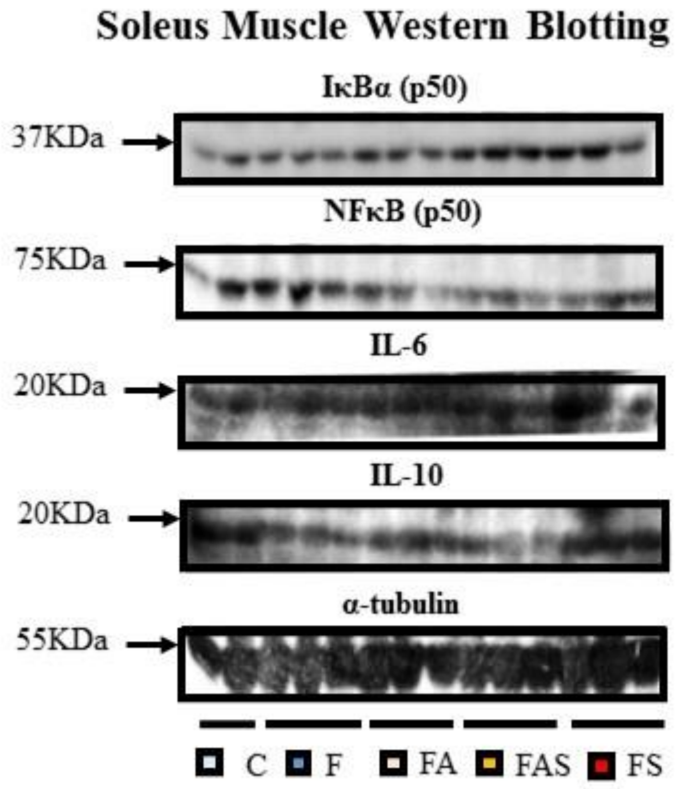

Gels have been run at same conditions. For IκB-α, NF-κB (p50), IL-6, IL-10 and α-tubulin. Western blotting signal was detected by special films and later scanned for density quantification. C: Control; F: Fructose; FA: Fructose Aerobic; FAS: Fructose Combined; FS: Fructose Strength.
